# Pathogenetic Mechanisms of Hypertension–Brain-Induced Complications: Focus on Molecular Mediators

**DOI:** 10.3390/ijms23052445

**Published:** 2022-02-23

**Authors:** Tiziana Di Chiara, Alessandro Del Cuore, Mario Daidone, Stefania Scaglione, Rosario Luca Norrito, Maria Grazia Puleo, Rosario Scaglione, Antonio Pinto, Antonino Tuttolomondo

**Affiliations:** Department of Health Promotion, Maternal and Infant Care, Internal Medicine and Medical Specialties, “G. D’Alessandro”, University of Palermo, Piazza delle Cliniche n.2, 90127 Palermo, Italy; tiziana.dichiara@unipa.it (T.D.C.); alex.delcuore@gmail.com (A.D.C.); mariodaidone@gmail.com (M.D.); stefaniascaglione95@gmail.com (S.S.); rosario94.norrito@gmail.com (R.L.N.); dott.ssamgpuleo@gmail.com (M.G.P.); rosario.scaglione@unipa.it (R.S.); antonio.pinto@unipa.it (A.P.)

**Keywords:** hypertension, cerebral complications, endothelial dysfunction, oxidative stress, neuroinflammation, innate immune system, Toll-like receptors

## Abstract

There is growing evidence that hypertension is the most important vascular risk factor for the development and progression of cardiovascular and cerebrovascular diseases. The brain is an early target of hypertension-induced organ damage and may manifest as stroke, subclinical cerebrovascular abnormalities and cognitive decline. The pathophysiological mechanisms of these harmful effects remain to be completely clarified. Hypertension is well known to alter the structure and function of cerebral blood vessels not only through its haemodynamics effects but also for its relationships with endothelial dysfunction, oxidative stress and inflammation. In the last several years, new possible mechanisms have been suggested to recognize the molecular basis of these pathological events. Accordingly, this review summarizes the factors involved in hypertension-induced brain complications, such as haemodynamic factors, endothelial dysfunction and oxidative stress, inflammation and intervention of innate immune system, with particular regard to the role of Toll-like receptors that have to be considered dominant components of the innate immune system. The complete definition of their prognostic role in the development and progression of hypertensive brain damage will be of great help in the identification of new markers of vascular damage and the implementation of innovative targeted therapeutic strategies.

**Table of Contents**
**Abstract**
**1. Introduction**
1.1. Blood Pressure Variability and Brain Damage
1.2. Hypertension and Stroke
1.3. Hypertension and Cerebral Small Vessel Diseases
1.4. Hypertension, Dementia and Progression of Brain Damage
**2. Pathogenetic Mechanisms of Hypertension–Brain-Induced Complications: Traditional Mechanisms**
2.1. Cerebral Blood Flow Autoregulation
2.2. Endothelial Dysfunction and Oxidative Stress
2.3. Mitochondrial Dysfunction
2.4. Microcirculation
2.5. Endothelial Activation and BBD Involvement
**3. Pathogenetic Mechanisms of Hypertension–Brain-Induced Complications: New Factors**
3.1. Role of Neuroinflammation
3.2. Role of the Innate Immune System
3.2.1. TRLs: Discovery, Structure and Function
3.2.2. Mechanisms of DAMPs Presentation
3.2.3. TLRs and Brain Damage-Related Hypertension
3.2.4. The Potential Therapeutic Role of TLRs in Cardiovascular Disorders
**4. Conclusions**

## 1. Introduction

The brain is an early target of hypertension-induced organ damage that can manifest itself either in the acute form such as thrombotic, embolic or haemorrhagic stroke or in the chronic form such as vascular dementia and cognitive impairment [1,2]. In addition, hypertension-related small vessel disease can contribute to the occurrence of lacunar infarction, alterations of the white matter and intracerebral haemorrhagic events both to the development of vascular dementia and influence on Alzheimer’s pathology, lowering the threshold at which signs and symptoms occur. Accordingly, hypertension is increasingly recognized as a “global neurological problem” [2]. 

There is growing evidence that hypertension represents the most important modifiable risk factor, after age, for haemorrhagic and ischemic stroke and cerebral small vessel disease (cSVD). Moreover, long-lasting hypertension can induce the development of cognitive impairment and dementia and influence its progression, even if both are dependent on old age. Despite the relationship among aging, hypertension and cognitive function being supported by complex and not yet fully identified mechanisms, blood pressure can, to date, be considered a marker of “cerebrovascular health” [3].

Several factors are able to increase the individual predisposition to the cerebrovascular consequences of arterial hypertension, so their identification is essentially better to define the relationship between blood pressure and brain functions. In assessing brain damage induced by arterial hypertension, particular importance has now been given to certain characteristics of hypertension, such as blood pressure variability.

### 1.1. Blood Pressure Variability and Brain Damage

Consistent data indicate that the increase in systolic and diastolic blood pressure and the increase in blood pressure variability is associated with organ damage and with morbidity and mortality from cerebrovascular and cardiovascular events in hypertensive subjects. Blood pressure is typically characterized by both short-term variability over 24 h (beat-to-beat, minute-to-minute, hour-to-hour and day-to-night changes) and long-term variability over more extended periods (days, weeks, months, seasons and even years) [4,5]. These variations may depend on the interplay of environmental and behavioural factors, as well as on innate changes in cardiovascular regulatory mechanisms. Blood pressure variability increases with age and is greater in women, diabetics, smokers and patients with peripheral vascular disease, atrial fibrillation or previous TIA or stroke [5]. Therefore, recent studies have identified the role of blood pressure variability as an independent risk factor for the progression of organ damage related to hypertension, including brain damage and cognitive impairment [6]. However, it is not known whether increased blood pressure variability is the cause or result of organ damage and should be considered as one of the new targets of hypertension treatment [4,7,8,9].

The changes in blood pressure variability are attributable to various factors, including vascular, neural and humoral factors, and they are reported in Table 1 [8]. Vascular factors may be the leading cause of worsening of blood pressure variability in hypertensive patients, especially in elderly patients, in patients with a long history of hypertension and patients with advanced atherosclerosis [8,9]. 

Some experimental studies indicate that the activation of the local Angiotensin II (Ang II) and mineralocorticoid receptors represent the molecular mechanism by which the increase in blood pressure variability worsened organ damage in hypertensive patients. Currently, the initial mechanism by which an increase in blood pressure variability activates the local Ang II and the mineralocorticoid receptors remains undetermined. However, stress on the vessel wall can be the main effect of increasing blood pressure variability, and, therefore, its normalization could represent a strategic goal in preventing target organ damage related to hypertension [4].

### 1.2. Hypertension and Stroke

The relationship between stroke and arterial hypertension is part of the history of scientific research of the last 70 years, the subject of countless pathophysiological studies and clinical trials that have addressed the epidemiology of stroke and hypertension, pathophysiology and above all, the effects of the management of hypertension on stroke outcomes. Furthermore, over 50% of strokes are attributable to hypertension [10], so the recommendations of the guidelines of the most important scientific societies in this field suggest that stroke prevention represents one of the most important goals of the management and treatment of arterial hypertension [1,10,11,12].

**Figure 1:**
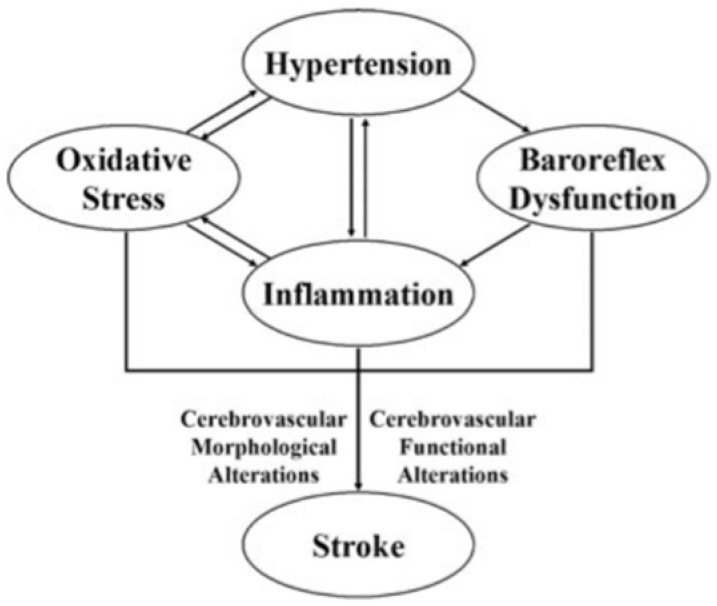
Pathogenetic links between hypertension and stroke (from [12], modified).

Cerebral stroke is the typical clinical manifestation of the rapid loss of brain function due to the lack of blood supply to the brain due to thrombosis or embolism (ischemic stroke) or haemorrhage (haemorrhagic stroke). It is well known that there is a delay between the onset of hypertension and a hypertensive complication. A series of changes occur in the cardiovascular system during this long period, including cerebral circulation. These changes, such as vascular remodelling, inflammation, oxidative stress, baroreflex dysfunction, etc., may contribute to the pathogenesis of stroke in hypertension [12,13,14,15,16,17,18,19,20] (Figure 1). 

The complete understanding of the molecular basis of the pathogenetic mechanisms involved in hypertension-induced stroke is of great help in developing new preventive strategies, especially providing new therapeutic targets.

### 1.3. Hypertension and cSVD

cSVD includes a group of pathological processes with various aetiologies affecting the brain’s small arteries, arterioles, venules and capillaries and is one of the leading causes of stroke and vascular and mixed dementia [2,21].

There are different types of sporadic or hereditary cSVD, and among them, the form linked to arteriolosclerosis, called type 1, is mainly related to hypertension, affecting the kidney and retina. Type 1 cSVD is characterized by loss of vascular smooth muscle cells (VSMCs), deposits of fibroyaline material, narrowing of the lumen and thickening of the vessel wall [2,21,22]. 

The effects on the brain parenchyma of cSVD, both sporadic and hereditary, are represented by lesions located mainly in the subcortical structures and include lacunar, ischemic infarcts, white matter lesions and intracerebral haemorrhage. Therefore, the clinical manifestations of cSVD are characterized by a wide range of symptoms, including typical signs of stroke and neurological deficits ranging from mild to progressive cognitive decline, dementia, depression and disability [2,21,22]. The mechanisms linking cSVD to parenchyma damage, both ischemic and haemorrhagic, are heterogeneous and not fully understood. They include chronic hypoperfusion, acute occlusion of the vessels, damage to the blood-brain barrier (BBB), local subclinical inflammation and apoptosis of oligodendrocytes. Alterations of the vessel wall can be responsible for the rupture of the vessel, thus manifesting itself as haemorrhagic cSVD, or for the stenosis of the vessel lumen or its functional dysregulation, inducing chronic hypoperfusion, being, in turn, responsible for an incomplete stroke or acute focal necrosis (lacunar stroke). These mechanisms interact with the changes induced by damage to the endothelium, as reported in the following part of this review.

### 1.4. Hypertension, Dementia and Progression of Brain Damage

Particular attention has recently been given to the role of hypertension on end-stage brain damage associated with a decline in cognitive function. Loss of cognitive function is one of the most devastating manifestations of aging and the progression of vascular diseases.

Cognitive decline is fast becoming a major cause of disability worldwide and contributes significantly to increased mortality [23]. Results from cross-sectional and longitudinal studies [24,25,26,27] show that hypertension plays a primary role in the onset of brain disease in small and large vessels resulting in brain damage and dementia. A reduction in cerebrovascular reserve capacity and the emergence of degenerative changes in the vascular wall underlies complete and incomplete cerebral infarcts, haemorrhages and white matter hyperintensity. In addition, it has been reported that the increase in systolic blood pressure correlates linearly to some markers of microstructural damage of the cerebral white matter in healthy young adults affecting various brain areas [28]. This damage can lead to what is known as “Unsuccessful aging”, characterized by impairment in specific cognitive domains such as those serving language, executive functions and visuospatial memory [29,30,31]. 

Hypertension can also lead to white matter lesions, accelerating the progression of arterial aging measured as arterial stiffness. This observation suggests that the effects of hypertension go beyond those traditionally associated with stroke and that early preventive strategies are essential to stem chronic brain damage. Although it is not clear whether this brain damage is reversible or can be slowed down by using antihypertensive treatments, early control of blood pressure in young subjects is recommended to avoid small myelin lesions and decreased cognitive performance [32].

Conversely, contradictory results have been reported for the older age groups, in which aggressive antihypertensive treatment does not appear to be associated with a subsequent favourable cognitive outcome [33]. Therefore, it is still uncertain whether adult hypertension is a risk factor for cognitive impairment in old age or whether cognitive decline and dementia are linked to hypertension only if it is long-lasting and/or develops in youth [34]. Therefore, the protective effect of antihypertensive therapy against dementia and stroke-related cognitive decline in the elderly is still debated, as a certain level of blood pressure appears to be necessary for the elderly to maintain cerebral perfusion and preserve cognitive abilities [29,33,34,35]. 

Although the hemodynamic effects of high blood pressure are undoubtedly the main factor contributing to brain damage, in recent years, several studies have identified some mediators, including molecular ones, crucially involved in hypertensive brain damage. 

This review is structured in two parts. In the first, we analyse the traditional pathogenetic mechanisms of brain complications induced by arterial hypertension, particularly the effects of hypertension on cerebral autoregulation blood flow, endothelial function and oxidative stress; microcirculation; and the BBR. 

In the second part, we focus on the new mechanisms recently proposed as the molecular links between hypertension and brain damage, such as the mediators of neuroinflammation and the intervention of the innate immune system.

## 2. Pathogenetic Mechanisms of Hypertension–Brain Induced Complications: Traditional Mechanisms

Several mechanisms are traditionally involved in the pathogenesis of hypertensive cerebral damage, including cerebral blood flow autoregulation, endothelial and mitochondrial dysfunction and oxidative stress. Furthermore, the development and progression of hypertensive brain damage can be conditioned by platelet activation, collagen turnover, alterations in coagulation, fibrinolysis, arterial stiffness, sympathetic hyperactivity or remodelling of the extracellular matrix, as well as from several hormone systems, including the renin–angiotensin–aldosterone system [36,37]. All these processes have one element in common: an increase in oxidative stress [16,36,37].

### 2.1. Cerebral Blood Flow (CBF) Autoregulation

**Figure 2:**
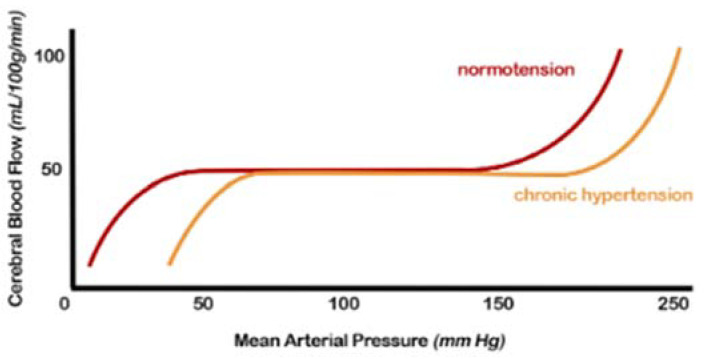
Cerebral autoregulation curve: in patients with hypertension, this range is “right-shifted”, or in other words, the normal range mean arterial pressure in which cerebral blood flow remains constant due to cerebral autoregulation is higher. Both the lower and upper limits are shifted (from [7], modified).

Regulating CBF in a normal brain ensures relatively constancy over a wide range of blood pressure changes [38]. Autoregulation is impaired when blood pressure exceeds the compensatory vasoconstrictor or vasodilatory capacity. This event usually occurs when the average blood pressure falls below 50 mm Hg or rises above 150 mm Hg in a normotensive subject (Figure 2). Experimental and clinical studies on the cerebral circulation have shown that arterial hypertension alters the autoregulation curve towards the right, i.e., higher-pressure values. The upper limit of self-regulation can be increased up to 30 mmHg. At the same time, the lower limit at which adequate CBF can be maintained is shifted to the right with the result that the symptoms of cerebral hypoperfusion develop at a correspondingly higher blood pressure level than normotensive ones.

Furthermore, in the presence of hemodynamic compromise, vasodilation of the intracranial arterial vessels is observed. This underlying vasodilation limits the brain’s ability to further vasodilate in response to other ischemic events, resulting in an increased risk of subsequent accidents. Hypertensive patients show an altered cerebrovascular reserve, which can be corrected by appropriate antihypertensive therapy [38,39].

The exact mechanisms by which hypertension affects autoregulation of CBF are not fully understood but likely include alterations in myogenic tone and remodelling of internal vessels, increasing the wall-to-lumen ratio [7,12,29,38,39].

The cerebral autoregulatory response occurs primarily in the small vessels and has both myogenic and neurogenic components. Myogenic autoregulation occurs in the smallest arterioles and appears to be primarily responsible for cerebral autoregulation at blood pressure above the normal physiological range. On the other hand, neurogenic autoregulation acts at the level of large arterioles and small arteries by the autonomic perivascular sympathetic nerves. Endothelial dysfunction also causes excess nitric oxide (NO) production via upregulation of NO synthase. NO induces vasodilation, particularly in conditions of high intravascular flows, which would tend to counteract the protective effects of vasoconstriction. Some studies have also shown that NO can increase the permeability of cerebral vessels through a mechanism controlled by cyclic guanosine monophosphate (cGMP) [39,40].

Studies using angiotensinogen knockout mice [41] have identified the role of Ang II in cerebral artery remodelling by reducing wall thickness, and wall-to-lumen ratio in elderly spontaneously hypertensive rats (SHRs) treated with angiotensin-converting enzyme (ACE) inhibitor [42]. In addition, Ang II and aldosterone have been linked to the production of reactive oxygen species (ROS), which are known to represent key mediators of cerebrovascular dysfunction in arterial hypertension, as they contribute to vasorarefaction (loss of arterioles and capillaries) and the structural remodelling of cerebral blood vessels, resulting in chronic hypoperfusion of the brain. Hypertension also increases the permeability of the BBB via ROS and impairs its ability to regulate central nervous system homeostasis [15,43]. ACE inhibition may also attenuate the increased permeability of the BBB associated with hypertension [42].

### 2.2. Endothelial Dysfunction and Oxidative Stress

The healthy endothelium is characterized by several protective properties against atherosclerosis in general, through the regulation of vascular tone, the antioxidant and anti-inflammatory effects, the inhibitory effects on the adhesion and migration of leukocytes and VSMCs and the inhibitory effects on platelet aggregation. 

The endothelium is composed of a single layer of cells that covers the internal surface of the blood vessels; it is considered a dynamic organ that acts as a natural barrier between blood and the vessel wall and has a wide variety of roles in controlling vascular function. As a consequence of alterations in endothelial function, various mechanisms are triggered, which determine vascular alterations. Endothelial cells (ECs) are the primary regulator of vascular homeostasis due to their interaction with circulating cells and VSMCs. In addition, they modulate blood flow, control plasma permeability and influence adhesion and aggregation of platelets and leukocytes [44,45,46]. 

The main pathways involved in the main endothelial functions are reported in Table 2, and they include regulation of vascular tone, fibrinolysis and coagulation, inflammation and formation, repair and remodelling of blood vessels.

These endothelium functions, even if they are described separately, are closely interrelated.

The term endothelial dysfunction identifies the transition from a normal to a damaged endothelium that can express itself with a proinflammatory, constricting, proliferative and procoagulative phenotype. Endothelial dysfunction has been studied extensively in peripheral arteries and was found to precede the elevation in blood pressure and, once developed, to correlate with its severity and target organ damage, including brain damage [15,16,44,47,48,49].

The main factors underlying endothelial dysfunction are therefore represented by reduced bioavailability of NO, impaired smooth muscle response to vasodilators, increased production of vasoconstrictive substances or high shear stress [15,16,36,37,44,48]. As previously reported in the introduction section, the endothelium dysfunction is accompanied by increased production of NO supported by alterations in NO metabolism (high NO degradation, NO inactivation or the presence of NO inhibitors) and with a consequent increase in oxidative stress. Oxidative stress is a condition in which ROS production exceeds the capacity of the antioxidant defence system. Excessive ROS generation, reduced antioxidant capacity or a combination of both can lead to oxidative stress. Persistent oxidative stress can deplete the effectiveness of antioxidant molecules, inactivate the enzymes responsible for antioxidant action and, therefore, compromise the cellular defence system [49,50].

There is compelling evidence that oxidative stress plays a critical part in the pathogenesis of hypertension and stroke as a long-term complication [15,16,48,49,50]. The increase in oxidative stress at the cellular level causes oxidative damage, altering the structure of molecules such as deoxyribonucleic acid, some amino acids, proteins, lipids and carbohydrates [44,45,48].

A particularly important radical for cardiovascular biology is superoxide, which is formed by subtracting an electron from oxygen. Superoxide can act as an oxidant, a reducing agent and a progenitor of other ROS. The superoxide anion is the primary determinant of the synthesis and availability of NO with vasoconstrictive action, and its production is stimulated by Ang II and endothelin 1. The oxidative excess is also linked to a proinflammatory state of the vessel wall, which, in turn, reduces the bioavailability of NO [15,44,48,50].

It is now a consolidated fact from the results of several studies, as well as of our team, conducted over the last 30 years that endothelial dysfunction represents one of the early markers of atherosclerosis and that it is present in arterial hypertension, representing one of the early mechanisms for the onset of organ damage associated and, in particular, brain damage [15,16,36,37,39,48,49,50]. Arterial hypertension causes an increase in the turnover of ECs with a reduction in the endothelial capacity to produce endothelial releasing factors (ERF) and consequent vasoconstriction. In addition, in arterial hypertension, a clear reduction in NO levels and an increase in ROS is documented, which in turn, in addition to inducing vasoconstriction and vascular remodelling with a consequent increase in peripheral vascular resistance, can facilitate sodium and water retention. These alterations are known to participate in the pathogenesis of arterial hypertension and associated brain damage.

### 2.3. Mitochondrial Dysfunction

Several studies, both in vitro and in vivo, have emphasized the importance of mitochondrial dysfunction, as a source of increased ROS, in the pathogenesis of endothelial dysfunction and, consequently, of atherosclerosis [51]. The increase in ROS production in mitochondria induces modifications of some molecules, including lipids, proteins and mitochondrial DNA (mtDNA). The latter is the most sensitive molecule to ROS-mediated damage. VSMCs and ECs exposed to ROS cause a reduction in total cellular ATP pools through damage to mtDNA, as well as alterations in mtDNA-encoded mRNA transcripts [52].

Studies conducted in experimental models in animals infused with Ang II and in ECs exposed to Ang II [53,54] indicate the existence of a pathophysiological link between ROS, cytoplasmic and mitochondrial and endothelial dysfunction in hypertension. Infusion of Ang II would decrease the membrane potential, the cellular respiratory ratio and the content of low molecular weight thiols. These deleterious effects of Ang II on mitochondrial function have been associated with increased cellular superoxide production and a decreased endothelial bioavailability of NO [54].

This mechanism plays an essential role in the occurrence of cerebrovascular damage induced by hypertension. The alterations in energy production that occur in brain cells represent the basis for several brain alterations. Brain mitochondria produce about 90% of the energy used by brain cells [55], necessary for the performance of their functions, such as intercellular communication and the transmission of stimuli and signals. An adequate energy supply by the mitochondria is essential for neuronal excitability and survival, so they are implicated in the pathogenesis of neurodegenerative diseases and cerebral ischemia.

Recent data have suggested a close link between excessive ROS generation and the development of neuronal death [56]. The brain is particularly prone to oxidative damage, both due to the lack of antioxidant defences [56] and because it can detect targets of free radicals derived from oxygen, such as the high traffic of calcium through neuronal membranes, presence of excitotoxic amino acids and auto-oxidizable neurotransmitters and a high quantity of polyunsaturated fatty acids contained within membrane lipids. 

ROS contribute not only to the damage of macromolecules but also to the transduction of apoptotic signals. Overproduction of ROS by the respiratory chain of brain mitochondria can progressively impair mitochondrial energy metabolism in hypertension. This phenomenon may be implicated in the vulnerability to cerebral ischemia, resulting in progressive neuronal cell death [55].

The experimental evidence linking mitochondrial dysfunction with cerebral vascular damage in hypertensive is limited. However, the evidence that an assembly defect in mitochondrial complexes I and V has been demonstrated in the brain mitochondria of SHRs seems interesting [57]. To better understand the consequences of these data, it is necessary to consider the functions and the energetic cellular role of mitochondrial complexes I and V.

Mitochondrial complex I, or nicotinamide adenine dinucleotide phosphate (NADPH) ubiquinone oxidoreductase, is the most important of the mitochondria mediate oxidative phosphorylation (OXPHOS), contributing about 40% to the energy required for the synthesis of adenosine triphosphate (ATP) by ATP synthase [58]. On the other hand, mitochondrial complex V is responsible for the catalytic phosphorylation of adenosine diphosphate (ADP) to form ATP. Therefore, as a consequence of the assembly defects reported, a reduction in the production of ATP is present in the brains of the SHRs, which induces cellular energy deficiency [57].

It is also known that mitochondria contribute to the intracellular homeostasis of the calcium ion [59]. Some studies indicate that, in elderly SHRs, it is possible to document a reduction in the potential of the mitochondrial inner membrane and consequent impairment of the activity of calcium-dependent enzymes, such as mitochondrial isoform of nitric oxide synthase (mtNOS). These alterations can undoubtedly contribute to a progression of mitochondrial dysfunction and neuronal cell death during hypertension [59]. It is interesting to note that mtNOS, located within the mitochondrial membrane, is involved in cell apoptosis by modulating the transmembrane potential, inhibiting chaim respiration and ATP synthesis, and is correlated with the onset of brain damage induced by hypertension [60].

### 2.4. Microcirculation

In the course of arterial hypertension, it is possible to document alterations in the cerebral microcirculation consisting of functional alterations that influence the vasomotor capacity, usually capable of transiently modifying the blood flow, which sometimes leads to thrombotic episodes [2,7,10,12,13,32,39,61].

Critical deficits in global or regional brain perfusion induce suppression of brain activity and cognitive dysfunction [62]. Inflammation can also damage the vessel wall through alterations in endothelial function that result in a reduced function of the cerebral microcirculation with irreversible neuronal damage [63]. Cerebral microbleeds associated with hypertension are typically localized in the basal ganglia, thalamus, brainstem and cerebellum, while a lobar distribution linked to cerebral amyloid angiopathy is common [64]. Functional impairment of the endothelium appears to be an early indicator of vascular dysfunction in cSVD and large cerebral vessels [44,48,65]. Unfortunately, direct assessment of cerebral endothelial function in humans is not feasible, limiting the number of studies available. A recent study analysed cerebral and peripheral vessel reactivity measurements in response to CO_2_ inhalation using transcranial doppler and duplex ultrasound in lacunar stroke patients and control subjects. Abnormalities in peripheral artery reactivity appeared to be related to vascular risk factors, and the severity of endothelial dysfunction in the cerebral arteries was related to the onset of lacunar stroke in cSVD patients [66]. It has also been shown that endothelial function, assessed by NO metabolism analysis, is impaired in patients with lacunar infarction and associated cSVD [67]. 

As previously reported, ROS are key mediators of cerebrovascular dysfunction in hypertension, as they contribute to vascular rarefaction and structural remodelling of cerebral blood vessels and, therefore, lead to functional alterations with profound consequences for CBF. In particular, experimental studies, including models of hypertension in rodents, report that targeting the enzyme that produces ROS, NADPH oxidase or its assembly protects against cerebrovascular oxidative stress and, consequently, from alterations endothelium-dependent relaxation and functional hyperaemia [68,69]. However, existing studies showing the direct effect of hypertension on cerebral arterial tone [70] mainly describe endothelial dysfunction in isolated arteries; only a few studies show a direct link between endothelium-dependent vasodilation and CBF. In addition, postmortem histological findings, conducted in patients with severe cSVD, show in these subjects an intact endothelial layer in the small arteries and the frontal white matter, while an apparent loss of myocytes and other wall cells was documented [71].

These conflicting aspects require further studies in order to document the role of endothelial dysfunction of small cerebral arteries exposed to high blood pressure before and during overt cSVD.

### 2.5. Endothelial Activation and BBB Involvement

The endothelium exhibits a high degree of structural and functional heterogeneity within the vascular tree. In the central nervous system, the so-called “neurovascular unit” refers to the possibility of a connection between microvessels and the function of neurons and the responses of these compartments to injury. The “neurovascular unit” consists of microvessels, astrocytes, neurons and their axons and other supporting cells that can modulate the unit’s function [72]. ECs in brain microvessels are part of the BBB and constitute a highly specialized phenotype. This barrier allows a rigorous control of the exchange of solutes and circulating cells between the plasma and the interstitial space. In most brain regions, cerebral ECs are connected by junctions that function as a natural “physical barrier” preventing molecular traffic between the blood and the brain [35]. BBB also acts as a “transport barrier”, supported by specific systems regulating the transcellular trafficking of small hydrophilic molecules, as well as a “metabolic barrier”, given the presence of both intracellular and extracellular enzymes [73].

Regarding the role of BBB in cSVD, it is necessary to consider the different types of associated brain lesions and that the components mentioned above are closely related to each other. Presumably, endothelial dysfunction is one of the main determinants of cerebral vessels’ structural and functional alterations. As we reported earlier, the imbalance between the production of vasodilator and vasoconstrictor molecules as well as the haemodynamic effects induced by hypertension lead to endothelial activation, a prerequisite for thrombo-inflammation and an early indicator of impaired BBB, which is usually accompanied by an increase in circulating adhesion molecules which condition the rolling, adhesion and migration of leukocytes [74]. Peripheral markers of endothelial activation include soluble vascular cell adhesion molecules-1 (sVCAM-1), soluble intercellular adhesion molecule-1 (sICAM-1), sP-selectin and sE-selectin, which are associated with cSVD markers and a reduction in cognitive performance in hypertensive patients. This fact indicates an essential role of endothelial activation in the pathogenesis of cSVD mediated by hypertension [75].

Levels of sICAM-1 appear to be an important marker for lacunar stroke and early neurological deterioration [67], even if its origin is not exclusively endothelial, which limits its specificity. More specific endothelial activation markers have emerged recently, and among them, sE-selectin seems to correlate with the number of microbleeds in patients with cSVD [76]. Other studies have shown an increase in adhesion molecules, the rolling of leukocytes along the cerebral vessels and the infiltration and accumulation of T cells in the perivascular spaces in hypertensive individuals, suggesting a causal link between endothelial activation and cerebrovascular dysfunction [77,78].

Other data indicate the presence of a thrombo-inflammatory state in elderly hypertensive subjects with cSVD. Thrombo-inflammation favours vascular occlusions, resulting in greater permeability of the BBB and triggering damage to the vessel wall with consequent vascular ruptures [79]. It has also been described that impairment of BBB resulting from alterations of its cellular components occurs during neurodegenerative diseases [80] but has also been associated with cSVD and lacunar infarcts [81]. However, it still remains uncertain whether BBB dysfunction is the main cause of the changes occurring in the cerebral microcirculation, since, to date, studies have only reported an increase in BBB permeability in cSVD associated with arterial hypertension [82,83,84]. Nevertheless, the simultaneous presence in hypertensive subjects of an increased number of activated circulating immunocompetent cells, of endothelial activation and the presence of microglial activation in the central nervous system indicates the existence of a causal relationship between elevated arterial pressure and impaired BBB [2,7,29,35]. 

## 3. Pathogenetic Mechanisms of Hypertension—Brain Induced Complications: New Factors

The pathogenetic mechanism of brain damage induced by arterial hypertension has long been identified in the mechanical effect of increased hydrostatic pressure on vessel walls. However, new possible mechanisms have been identified that can help to clarify the molecular basis of this pathological event. Over the past ten years, several studies have demonstrated the role of neuroinflammation, defined as inflammation in response to insults derived from various pathogenic noxae linked to various pathologies, such as ischemic stroke, in the occurrence and progression of brain damage [82,83]. In such cases, neuronal death and apoptosis of brain cells activate an inflammatory cascade driven by chemokines and cytokines, which, together with the intervention of some cells of the innate immune system, are responsible for a further extension of brain damage that can lead to cognitive disability [82,83,84,85].

### 3.1. Role of Neuroinflammation

The inflammatory response to stroke generally develops as a consequence of two sequential but closely related phenomena:

1. The activation of microglia and perivascular and parenchymal resident macrophages;
2. The infiltration of peripheral inflammatory cells into the brain.

Once activated, the glial cells can produce a large variety of proinflammatory mediators suitable for damaging the cerebral endothelium, which, in addition to constituting the BBB, is involved in the determinism and the modulation of the cerebral inflammatory response [84,85].

For example, releasing proinflammatory vasoactive mediators by the cerebral endothelium during the acute phase of stroke can induce blood clotting in the microcirculation, extending the infarct area. Furthermore, once the “neuroinflammatory” response induced by ischemic or haemorrhagic stroke is triggered, it progresses for several days after the onset of symptoms, contributing to the pathogenesis of the later stages of brain damage and causing a worsening neurological prognosis. These considerations support new therapeutic strategies for stroke for which a deepening of knowledge of the neuroinflammatory process is needed [86,87]. A variety of mediators involved in all stages of neuroinflammation, especially in ischemic stroke, have recently been reported from its initial stage to resolution. They include tumour necrosis factor-alfa (TNF-α), interleukin (IL) 1α, IL-1β, chemokine (C-X-C motif) ligand 7 (CXCL7), chemokine (C-C motif), ligand 5 (CCL5), chemokine (C-X-C motif) ligand 4 (CXCL4), chemokine (C-X3-C motif) ligand 1 (CX3CL1), adhesion molecules, proteases, prostanoids and leukotrienes, while IL-1, IL-10, TNF-α, IL-6, IL-20, IL-17, NADPH oxidase, chemokine (C-X-C motif) ligand 8 (CXCL8), inducible nitric oxide synthase (iNOS) and cyclooxygenase-2 (COX-2) are involved in the enhancement phase of this process. In addition, other molecules, including transforming growth factor β (TGF-β), IL-17, IL-10 and IL-23, mediate the resolution phase [18,19,88,89,90,91,92,93].

Finally, some molecules such as the neutrophil-to-lymphocyte ratio family pyrin domain-containing 3 (NLRP3) inflammasome, Dickkopf WNT Signalling Pathway Inhibitor 3 (DKK-3), dectin-1, MKEY and microRNAs (miRNAs), as well as being implicated in the course of neuroinflammation [18,19,86,88,89,90,91,92,93], have shown to have interesting characteristics that can attribute a target role in the management of brain damage, and they are summarized as follows:

- The NLRP3 inflammasome is responsible for the activation of IL-1β and the release of IL-18, which phosphorylate insulin receptor substrate 1 (IRS-1), worsening insulin resistance and causing neuronal death. The NLRP3 inflammasome is one of the primary mediators contributing to the neuroinflammation process and consequent brain damage [88].
- DKK-3 concentrations are associated with endothelial dysfunction and atherosclerosis. High or low levels of DKK-3 are able of inducing a worsening of outcomes after ischemic stroke [89].
- The interaction between Dectin-1 and damage-associated molecular patterns (DAMPs) determines the phosphorylation of immunoreceptor tyrosine-based activation motifs (ITAMs) and, subsequently, of spleen tyrosine kinase (SYK9), a kinase able to mediate the neuroinflammatory cascade through the release of some cytokines. Therefore, the inflammatory pathway mediated by Dectin-1/SYK plays a fundamental role in postictal neuroinflammation [90].
- The heterodimer CXCL4-CCL5 plays a crucial role in developing brain damage [91].
- MKEY, a cyclic peptide synthesized in mice, can avoid the formation of the heterodimer CXCL4-CCL5, thereby limiting ischemic brain injury and improving neurological deficits [92].
- The expression of some miRNAs, such as 126, 124-3p, 30a and 16, is considerably elevated in patients with acute ischemic brain injury, even if they still cannot be successfully used in clinical routine for obvious reasons, above all being the high cost and their execution in highly specialized laboratories [93].

These mediators interact with several innate immune system cells, such as activated ECs can increase the expression of adhesion molecules and chemokines, and interact with immune cells during the inflammatory process, including brain involvement [18,19,37,39,44,47,48].

### 3.2. Role of the Innate Immune System

The innate immune system plays an important role in defence mechanisms, as well as concerning infections also in response to tissue damage. For example, dendritic cells, macrophages, natural killer T cells and Toll-like receptors (TLRs) are immune system components recently linked to organ damage resulting from arterial hypertension [94]. In this regard, among the various components of the innate immune system, TLRs are becoming a significant research target in the field of cardiovascular pathophysiology, including atherosclerosis, hypertension and stroke [95]. 

TLRs are essential members of the family of pattern recognition receptors (PRRs) which can recognize and respond to a large repertoire of conserved molecular patterns presenting on both exogenous invading substances, as pathogen-associated molecular patterns (PAMPs), and endogenous molecules, as DAMPs, from injured tissue or cells and released after hypoxia, trauma and cell death. Prolonged or excessive activation of TLRs on immune and vascular cells has been proposed to induce chronic low-grade inflammation, leading to endothelial dysfunction and subsequent cardiovascular disease.

The interaction of TLRs with their ligands leads to the activation of downstream signalling pathways that induce an immune response by producing proinflammatory cytokines, including ILs, TNFs, monokines, and chemokines, type-I interferon and other inflammatory mediators. The secretion of these cytokines is concomitant with their downstream inflammation biomarkers, such as IL-6, C reactive protein (C-RP) and fibrinogen. The upstream and downstream inflammatory biomarkers are closely associated with an increased incidence of cardiovascular events. However, there are also cytokines, particularly TGF-β, IL-10 and interferon -α (IFN-α), that downregulate inflammatory reaction and protect the host against atherogenesis, which is the inherent function of the innate immune defence system. In addition, the activation of TLRs can also evoke other biological mediators in a cell-dependent manner, including platelet activation factor, ROS and nitrogen species to orchestrate the progression of diseases [94,95,96]. 

**Figure 3:**
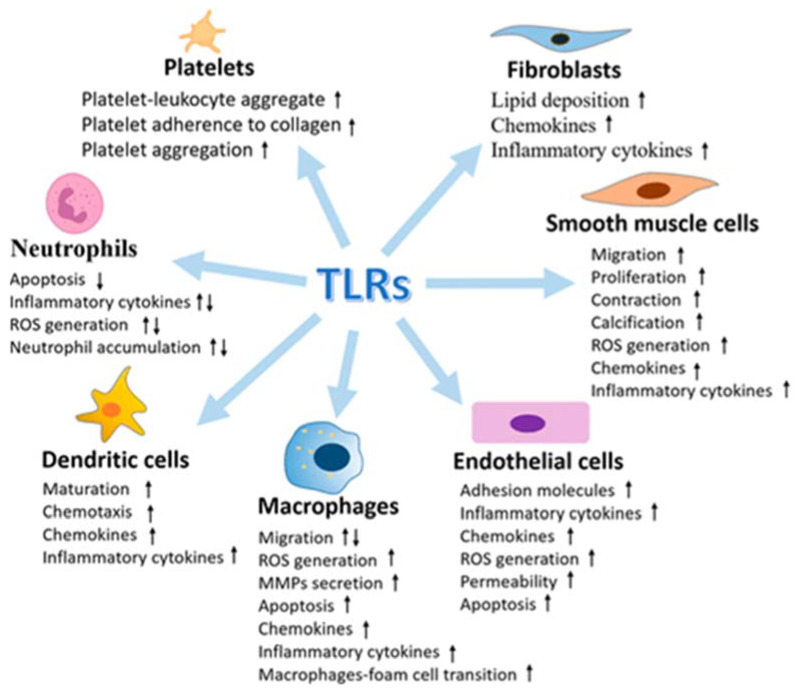
Multiple effects of Toll-like receptors (TLRs) in vasculature cells (from [96], modified).

Several examples have emerged to support the activation and contribution of TLRs to the progression of vascular atherothrombotic diseases. The vasculature system expresses all of the known human TLRs, and under vascular pathophysiology, the levels of TLRs are found to be upregulated (Figure 3).

#### 3.2.1. TRLs: Discovery, Structure and Function

The Toll receptors are named for their structural similarity to Toll, a receptor first discovered in the Drosophila melanogaster, through a mutation in the Toll gene, caused abnormal development [97]. The embryos carrying the mutation were named “Toll”; later, a human homolog more closely related to Drosophila Toll was cloned [98], and the “human Toll” was then renamed “TLR4”.

TRLs, as previously reported, are responsible for recognizing and initiating an inflammatory response to microbial components expressed by bacteria, fungi, protozoa and viruses, as well as to DAMPs released by dying cells or generated as a result of tissue injury and oxidation [99]. In addition, the low complexity of the TLR signal, which includes four adapter molecules and three downstream inflammatory transcription factors, represents an efficient means of upregulating proinflammatory genes [94,96,99]. 

Inflammatory genes expressed as a result of TLRs activation include cytokines, whose expression patterns drive the adaptive immune response (cell-mediated Th1 response or humoral/antibody Th2 response), chemokines (chemotactic cytokines) that drive cell migration to target tissues and cell adhesion molecules that promote binding, rolling and infiltration of immune cells into the vascular wall and translocation to end organs [100].

TLRs are expressed on specialized immune cells (e.g., macrophages and dendritic cells) and nonimmune cells (e.g., epithelial, fibroblast and ECs). 

TLRs are transmembrane proteins, or more specifically, type-I integral membrane glycoproteins with three structural domains:

(1) An amino (N)-terminal ectodomain that contains leucine-rich repeats and mediates ligand recognition;
(2) A single transmembrane domain that determines cellular localization;
(3) A carboxyl (C)-terminal cytoplasmic domain of the Toll/interleukin-1 receptor (TIR) that mediates downstream signalling.

So far, it has been shown that there are 10 TLR genes in humans (TLR1–TLR10) and 12 (TLR1–TLR9, TLR11–TLR13) in mice [101]. In general, TLRs can be divided into two groups based on their cellular location when detecting their respective ligands as follows:

- TLRs 1, 2, 4–6 and 11 are localized on the cell surface (cell surface TLRs) [94,96,102];
- TLRs 3, 7-9 and 13 reside in the intracellular compartments (intracellular TLRs) [94,96,102].

Cell surface TLRs respond to microbial membrane materials such as lipids, lipoproteins and proteins, while intracellular TLRs recognize nucleic acids. Collectively, TLRs provide rigorous surveillance of intracellular and extracellular compartments, detecting most viral and bacterial molecular signatures as well as host-derived molecules released from damaged and apoptotic cells.

Most TLRs are homodimeric, although some of them can form heterodimers. Ligand binding promotes engagement of the two TIR domains of the cytosolic site of each receptor, and as the TIR domains get closer, a new signalling platform is created [103]. The formation of this platform is required for the recruitment of adapters containing cytosolic TIR domains, a key step in the TLRs signal. Several transmembrane proteins play the role of co-receptors in the TLRs signal, and the ability of TLRs to cooperate with accessory proteins increases the range of ligands that TLRs can recognize. Among them, particular importance is attributed to the myeloid differentiation primary response gene88-dependent pathway (MyD 88) and the Toll/interleukin1 receptor domain-containing adapter protein (TIRAP) [98,99,102].

The recruitment of adapter proteins represents the initial phase of TLRs signal transduction and, consequently, the first step in activating the innate immune system. Indeed, TLR4 activates the pathways dependent on MyD88 and TIRAP, which lead to the production, respectively, of proinflammatory cytokines and type-I interferon. Various regulatory mechanisms are active in harmonizing the TLRs signal and avoiding an exaggerated immune response. Loss or deficiency of these regulatory mechanisms can be involved in developing immune-mediated and inflammatory diseases [94].

#### 3.2.2. Mechanisms of DAMPs Presentation

**Figure 4:**
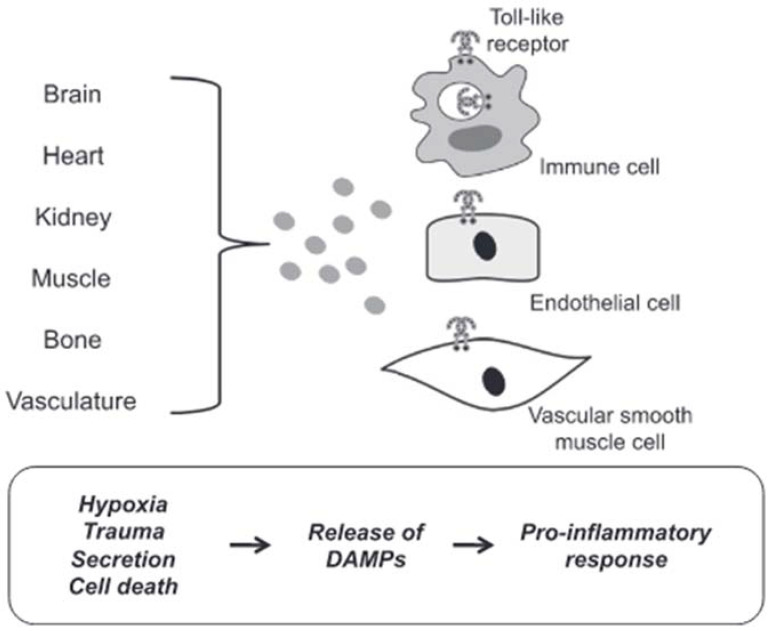
DAMPs induced activation of TLRs. Circulating DAMPs released after hypoxia, trauma and cell death lead to TLRs activation in immune cells, endothelial cells and vascular smooth muscle cells. Prolonged or excessive activation of TLRs on these cells provides a proinflammatory state, leading to endothelial dysfunction and subsequent cardiovascular disease (from [94], modified).

DAMPs are endogenous molecules that are usually contained within cell membranes and protected from exposure to components of the immune system. However, when a cell is stressed or its plasma membrane is damaged, these endogenous molecules can be expressed on cell surfaces or diffuse freely into the extracellular space. The immune system recognizes these molecules as a danger and induces a response [104]. In most cases in hypertensive subjects, cell death represents the triggering mechanism of inflammation induced by the release of DAMPs with consequent brain damage. Circulating DAMPs released after hypoxia, trauma and cell death result in activation of TLRs in vascular smooth muscle, immune and endothelial cells (Figure 4).

Prolonged or excessive activation of TLRs on these cells induces a proinflammatory state leading to endothelial dysfunction and subsequent cardiovascular disease [95]. Although there are many forms of cell death, necrotic cell death was generally thought to be the primary source of proinflammatory DAMPs due to the disintegration of the plasma membrane and the release of intracellular constituents [105]. Apoptosis can also be immunogenic due to the programmed release of immunostimulating molecules [106]. In this case, the release of DAMPs can be passive in the extracellular environment due to cell death or a damaged extracellular matrix, both active in the extracellular environment or on the surface of cells (i.e., neoantigens). Mechanisms of secretion and exposure are the results of cell stress. For these reasons, cell death would not seem to be the only precursor of the participation of DAMPs in the pathophysiology of cardiovascular diseases, in general, and of brain damage induced by arterial hypertension, in particular.

#### 3.2.3. TLRs and Brain Damage-Related Hypertension

The development and maintenance of arterial hypertension depend on the contribution of the kidneys, the autonomic nervous system and the vascular system. Uncontrolled immune system activation and inflammation have been proposed as a unifying mechanism between these three organs and systems. Therefore, TLRs represent potential candidates for mediating this aberrant inflammation [95].

TLR4 is the most studied and involved TLR in the aetiology of hypertension [95], based on the well-known association between TLR4 and angiotensin II, identified by some authors as DAMP [95,107] and demonstrated in various experimental models of hypertension, such as SHRs [108] or rats with angiotensin II-induced hypertension [109].

Conversely, although there is abundant literature supporting the role of TLR2 in atherogenesis, there are limited data to support the contribution of TLR2 in hypertension [94,95]. Nonetheless, TLR2 has been observed to mediate the dysfunction of several cell types that contribute to the development of hypertension. For example, in ECs, high-density lipoprotein from patients with chronic kidney disease can activate TLR2 (independent of TLR1 and TLR6) and reduce the bioavailability of NO, resulting in endothelial damage and inflammation and increased blood pressure [110]. Likewise, TLR5 has not been directly linked to hypertension but has been linked to metabolic syndrome, of which hypertension is one of the distinguishing factors. In particular, TLR5 knockout mice develop metabolic syndrome compared to wild control mice [111].

Furthermore, TLR7/8 and TLR9 induced in experimental studies produce the greatest proinflammatory responses to angiotensin II when triggered with the respective exogenous ligands [112]. 

Recently, it has been shown that TLR9 has an essential role in blood pressure regulation since, while its activation induces hypertension, its inhibition leads to a reduction in blood pressure in normotensive rats [113]. In addition, it has also been observed that TLR9 is a negative regulator of cardiac vagal tone and baroreflex function [114].

Overall, available data support the participation of TLRs in the aetiology of arterial hypertension and related organ damage, including brain damage. In this regard, it is known that cerebral ischemia causes an acute inflammatory reaction, which can exacerbate the brain damage caused by stroke [115]. The regulation of inflammation after an ictal event is multifactorial and includes vascular effects, distinct cellular responses, cell death and chemotaxis. Several cellular stipitis are involved in this process, including neurons, astrocytes, microglia and ECs, all of which respond to the resulting neuroinflammation in different ways [116]. TLRs are expressed on these brain cells and participate in the progression of brain damage through the following mechanisms:

(1) The stimulation of TLRs before the ischemic event is neuroprotective and preconditions the brain to tolerate hypoxia and nutrient deprivation [117];
(2) Postischemic activation of TLRs mediates neuroinflammation and neurodegeneration [94].

This role of TLRs has been demonstrated by the results of some experimental studies, which can be summarized as follows:

- Mice with TLR2 deficiency are protected against cell damage and death induced by ischemia [118,119,120];
- Ischemia causes an increase in the expression of TLR2 in neurons (118) and microglia associated with the lesion [119];
- The neurological damage and deficits caused by a stroke were significantly lower in TLR2-deficient mice compared to wild-type controls [118];
- Although acute ischemic lesions (24 to 72 h) have been observed to be smaller in TLR2-deficient mice, the subsequent innate immune response has been reported to be more pronounced, causing progression of the ischemic injury [120].

Although these data suggest a negative effect of TLR2 in stroke, other researchers have reported that preconditioning with a TLR2 ligand protected the brain from ischemia-reperfusion injury [121], possibly through a TLR2/protein kinase B mechanism-dependent signalling (TLR2/PI3K) [122]. Similarly, TLR4-deficient mice have been observed to have reduced ischemic damage [123], and a TLR4 antagonist is able to reduce neuroinflammation and neurological deficits after intracerebral haemorrhage [124]. On the other hand, TLR4-deficient mice showed less preconditioning-induced neuroprotection than wild-type controls [125]. This neuroprotective effect can be attributed to the ability of TLR4 to promote neurogenesis after stroke, inducing neuroblast migration and increasing the number of new neurons [126]. More uncertain data are reported on the role of TLR5 in the progression of brain damage, although some studies seem to indicate that TLR5 is involved in the postischemic neuroinflammatory response.

In regard to endosomal TLRs, it is possible to summarize the data from the literature as follows:

- TLR3 induces neuroprotection against ischemia through preconditioning [127];
- The expression of TLR7 and TLR8 is associated with a negative outcome with increased inflammatory responses in patients with acute ischemic stroke [128];
- TLR8 agonist induces increased neuronal cell death during oxygen or glucose deprivation, neurological deficit and T cell infiltration after stroke [129];
- TLR9 contributes to the progression of ischemic brain lesions [130], and the expression of TLR9 induces pronounced and dynamic changes predominantly in microglia [131];
- TLR9 activation induces neuroprotection against ischemic damage by increasing serum TNF-α by activating PI3K [132].

Overall, the data available suggest a double function of TLRs in cerebrovascular damage. Although they contribute significantly to neuroinflammation and immediate postischemic brain injury, controlled modulation of their signal can precondition individuals at risk for stroke and improve clinical outcomes, lesion size and neurological deficit.

#### 3.2.4. The Potential Therapeutic Role of TLRs in Cardiovascular Disorders

Molecular immunology and pathophysiology studies have provided new insights into the structural characteristics of TLRs, their ligands, their co-receptors and associated signalling proteins. TLR signalling triggers the transcriptional activation of different proinflammatory cytokines. Therefore, targeting the TLR signalling pathway is a plausible and complementary strategy to manage abnormal inflammation and vascular conditions. Therefore, both TLR agonists (inducers of protective immunity) and antagonists (suppressors of excessive inflammation) have been shown to have beneficial effects in various clinical conditions, such as cancer, microbial inflammation, autoimmunity and allergies, by modulating the tissue inflammatory response, while drug development targeting TLR regulation for cardiovascular disease is still in its infancy [94,96,98,99]. Nonetheless, new TLR inhibitory peptides have been identified capable of blocking the signalling of TLRs and suppressing the production of inflammatory cytokines [133], as well as mimetic peptides that seem to be able to increase stability, internalization and the sensitivity of the receptor, thus interrupting the interaction between TIR and MyD88 [134]. Furthermore, experimental data indicate that treatment with such peptides may protect against left ventricular dilation and hypertrophy in a mouse model of acute myocardial infarction [135].

However, designing efficient inhibitors is a challenging task that requires researchers to address issues such as toxicity, specificity and mode of administration. Peptidomimetics and peptide and protein inhibitors may be promising therapies targeting TLR adapters and disrupting TLR adapter interactions. Peptidomimetics are low molecular weight compounds that can easily translocate into the cell but often lack specificity. On the other hand, protein inhibitors induce a specific inhibition of the signal, but their size impairs their effective delivery into the cell. Administration of TLR inhibitors as purified proteins has been suggested, but the cost of this approach is currently prohibitive. Cell-penetrating portions, including short cationic peptides and fatty acids, have been successfully added to inhibitory peptides to cross the cell membrane and target the TLR signal [133]. The use of miRNAs to suppress excessive activation and inflammation of the immune system may hold promise, but safety, efficacy and specificity have not yet been addressed [93]. Accordingly, there is no compound in clinical trials that directly targets TLRs to treat vascular disorders to the best of our knowledge.

Nonetheless, novel TLR signalling modulators and devices are still being investigated [136,137,138,139,140,141,142,143,144,145,146], and it is foreseeable that, in the near future, there will be a TLR targeting clinical drug for the treatment of cardiovascular disease. 

## 4. Conclusions

The brain is one of the main target organs of arterial hypertension. It is responsible for a large share of the morbidity and mortality associated with this pathology, the most important risk factor for stroke and a nonsecondary determinant of cognitive decline. Despite the pathogenetic mechanism of brain damage induced by arterial hypertension being long-identified in the mechanical effect of increased hydrostatic pressure on vessel walls, new possible mechanisms have recently been identified that can help to clarify this event’s molecular basis, allowing the development of innovative specific and effective therapies.

Among them, particular interest has recently been addressed to the study of the role of TLRs with the demonstration that innate immunity provides essential contributions to the development of neuroinflammation and cardiovascular diseases, including arterial hypertension, which are now recognized as true chronic inflammatory diseases. However, some aspects of the mechanisms by which TLRs and the innate immune system contribute to the genesis and maintenance of cardiovascular diseases and brain damage induced by arterial hypertension remain to be clarified. Furthermore, the effects of the TLR signalling are known not to be limited to the activation of immune cells but also affect the function of resident vascular cells, raising the following questions:

- How can the TLRs signalling alter the different compartments of the blood vessels and the interaction between the layers of the vascular wall?
- How can the activation of TLRs modify the intracellular signal induced by the second messenger in vascular cells?

Another aspect of future research concerns the definition of the mechanisms through which TLRs mediate the interaction between innate immune memory and cardiovascular tissue dysfunction. Furthermore, it is unclear at what stage of cardiovascular disease, in general, and of arterial hypertension, in particular, the involvement of TLRs begins.

The resolution of these and many other questions make research in this field innovative, intriguing and promising, with great expectations both for the development of new biological and molecular markers capable of early identification of brain damage induced by arterial hypertension and also for the introduction of new therapeutic targets suitable for improving therapeutic strategies in this area.

## Figures and Tables

**Table 1:** Regulating factors of blood pressure variability.

Vascular Factors	Stiffness of arteries; compliance of resistant artery; vascular remodelling
Neural Factors	Sympathetic, parasympathetic and baroreflex system
Humoral Factors	Renin–angiotensin–aldosterone system; endothelin
Others	Stress, emotion, exercise, circadian rhythm, climate, environment

**Table 2:** Molecules produced and secreted by endothelial cells.

Regulation of Vascular Tone	(a) Vasodilation: nitric oxide, prostacyclin, endothelium-derived hyperpolarizing factors, adenosine
	(b) Vasoconstriction: endothelin-1, angiotensin II, thromboxane A2, reactive oxygen species
Balanced Blood Fluidity/Thrombosis	(a) Coagulation: heparin cofactor 2, factor V, protein S, protein C, thrombomodulin, tissue factor, von Willebrand factor
	(b) Fibrinolysis: tissue plasminogen activator, prostaglandins, plasminogen activator inhibitor type 1, urokinase
Vascular Inflammation and Immunological Process Control	(a) Cytokines: interleukins 1,6,8, monocyte chemoattractant protein-1
	(b) Adhesion Molecules: vascular adhesion protein 1, intercellular adhesion molecule 1, selectins
	(c) Growth Factors: Basic fibroblast growth factor, insulin-like growth factor, platelet-derived growth factor, transforming growth factor, tumour necrosis factor

## Abbreviations

ACE	angiotensin-converting enzyme
Ang II	angiotensin II
ADP	adenosine diphosphate
ATP	adenosine triphosphate
BBB	brain–blood barrier
CBF	cerebral blood flow
CCL5	chemokine (C-C motif) ligand 5
cGMP	cyclic guanosine monophosphate
CRP	C-reactive protein
cSVD	cerebral small vessel disease
CX3CL1	chemokine (C-X3-C motif) ligand 1
CXCL4	chemokine (C-X-C motif) ligand 4
CXCL7	chemokine (C-X-C motif) ligand 7
CXCL8	chemokine (C-X-C motif) ligand 8
COX-2	cyclooxygenase-2
DAMPs	damage-associated molecular patterns
DKK-3	Dickkopf WNT Signalling Pathway Inhibitor 3
ECs	endothelial cells
ERF	endothelial releasing factors
IL	interleukine
iNOS	inducible nitric oxide synthase
IFN-α	interferone-α
IRS-1	insulin receptor substrate 1
ITAMs	immunoreceptor tyrosine-based activation motifs
miRNAs	microRNAs
MyD88	myeloid differentiation primary response gene88-dependent pathway
mtDNA	mitochondrial DNA
mtNOS	mitochondrial isoform of nitric oxide synthase
NADPH	nicotinamide adenine dinucleotide phosphate
NLR	neutrophil-to-lymphocyte ratio
NLRP3	NLR family pyrin domain-containing 3
NO	nitric oxide
OXPHOS	mitochondria mediate oxidative phosphorylation
PAMPs	pathogen associated molecular patterns
P13K	protein kinase B mechanism-dependent signalling
PRRs	family of pattern recognition receptors
ROS	reactive oxygen species
SHRs	spontaneously hypertensive rats
SYK	spleen tyrosine kinase
TIR	Toll/interleukin-1 receptor
TIRAP	Toll-interleukin 1 receptor domain-containing adapter protein
TLRs	Toll-like receptors
TNF-α	tumour necrosis factor-alfa
TGF-β	transforming growth factor-beta
sICAM-1	soluble intercellular adhesion molecule-1
sVCAM-1	soluble vascular cell adhesion molecules-1
VSMCs	vascular smooth muscle cells
